# High seroprevalance of *Neospora caninum* in dogs in Victoria, Australia, compared to 20 years ago

**DOI:** 10.1186/s13071-017-2464-2

**Published:** 2017-10-19

**Authors:** Sarah Sloan, Jan Šlapeta, Abdul Jabbar, Jaimie Hunnam, Bert De Groef, Grant Rawlin, Christina McCowan

**Affiliations:** 1 Veterinary Pathobiology, Department of Economic Development, Jobs, Transport and Resources, Bundoora, VIC Australia; 2 Department of Physiology, Anatomy and Microbiology, School of Life Sciences, La Trobe University, Bundoora, VIC Australia; 3 Sydney School of Veterinary Science, Faculty of Science, The University of Sydney, Sydney, NSW Australia; 4 Faculty of Veterinary and Agricultural Sciences, The University of Melbourne, Werribee, VIC Australia; 5 Agriculture and Resource Division, Department of Economic Development, Jobs, Transport and Resources, Bundoora, VIC Australia

**Keywords:** *Neospora caninum*, Epidemiology, Neosporosis, ELISA, IFAT, Dog, Canid, Fox, Seroprevalence

## Abstract

**Background:**

Canids are definitive hosts of the apicomplexan parasite *Neospora caninum*, the leading cause of abortion in cattle worldwide. For horizontal transmission from canids to occur, oocysts of *N. caninum* must be shed by the definitive host into the environment of susceptible intermediate hosts such as cattle. The purpose of this study was to determine the prevalence of *N. caninum* in canids in Victoria, Australia’s leading dairy producing state.

**Results:**

*Neospora*-like oocysts were observed in 8% (18/234) of faecal samples from wild dogs, domestic dogs and red foxes from Victoria, Australia. However, none tested positive for *N. caninum* DNA using a quantitative PCR. In a separate sample population, blood sera from 483 domestic dogs were tested for anti-*N. caninum* antibodies using competitive ELISA. A subset of cELISA samples were re-tested using indirect fluorescence antibody test (IFAT). A seroprevalence of 29.8% (144/483; 95% CI: 11.7–47.8%) was calculated when using cELISA; whereas it was 32.9% (27/80; 95% CI: 15.8–51.8%) using IFAT. Potential risk factors were evaluated using univariable analyses and then assessed in separate multivariable models. Using ‘aged’ dogs as a reference, the seroprevalence of ‘adolescent’ and ‘adult’ dogs was 88% (*P* = 0.05) and 91% (*P* = 0.08), respectively, indicating seroprevalence increases with age. There was a 19% higher likelihood of infection in rural locations (*P* = 0.10) relative to urban areas. Jack Russell Terriers had a 22% higher risk of a cELISA-positive result (*P* = 0.05) regardless of geographical location, age or sex.

**Conclusion:**

These results demonstrate that exposure to *N. caninum* in domestic dogs is widespread in Victoria, although faecal oocyst shedding is infrequent. Our results indicate increased *N. caninum* seroprevalance status in dogs over the past two decades. The results imply that dogs get either exposed to the infected meat more frequently or that vertical dam to foetus transmission is more frequent than previously thought. Our study calls for re-evaluation of historical *N. caninum* seroprevalance studies, because the attitude to dog diet changes.

**Electronic supplementary material:**

The online version of this article (10.1186/s13071-017-2464-2) contains supplementary material, which is available to authorized users.

## Background

*Neospora caninum* is an obligate intracellular, tissue cyst-forming, coccidian parasite regarded as the leading cause of abortion in cattle worldwide [1, 2]. Canids - domestic and wild dogs (*Canis lupus familiaris*), grey wolves (*Canis lupus*), coyotes (*Canis latrans*) and dingoes (*Canis lupus dingo*) - are confirmed definitive hosts in which sexual reproduction of *N. caninum* occurs [3–5]. The life-cycle of *N. caninum* is maintained between canids and cattle, but there is often a concurrent sylvatic life-cycle between wild canids and herbivore species, particularly wild ruminants (e.g. deer) [5, 6].

Horizontal transmission is facilitated by the shedding of oocysts in the faeces of the definitive host which sporulate and are then infectious when ingested, the primary route of infection for canids [6–9]. Alternatively, carnivores may be infected by ingestion of tissue cysts in the flesh of non-carnivore species [9]. Infection in ruminants can occur vertically from the infected dam to her offspring, or horizontally via ingestion of *N. caninum* oocysts via the faecal contamination of food or water [10].

Very little is known about the impact of *N. caninum* in Victoria, Australia’s leading dairy region. Australia is a major producer of agricultural products worldwide and the value of beef and milk production in 2015 was valued at $11.5 billion and $4.7 billion, respectively [11, 12]. The economic impact of *N. caninum*-associated abortion in Australian cattle has been estimated to be more than $100 million per year [13]. A study of *N. caninum* infection in dogs from Melbourne, Victoria, found a seroprevalence of 5% [14], but nothing has been done since then to determine the seroprevalence of Victorian dogs.

The primary objectives of this study were to determine the prevalence of oocyst shedding and seroprevalence of *N. caninum* in canids in Victoria, Australia. Oocyst shedding was evaluated using faecal flotations coupled with molecular diagnostics. Seroprevalence was evaluated using the competitive enzyme-linked immunosorbent assay (cELISA) and indirect fluorescence antibody test (IFAT). The data obtained enabled us to compare and contrast our data with those reported from 1997 [14]. Faecal and serological analyses of canids can help to identify at-risk locations and animal characteristics, therefore aiding in prevention of the spread of *N. caninum* and reducing its economic impact on the beef and dairy industries.

## Methods

### Sample collection

**Fig. 1:**
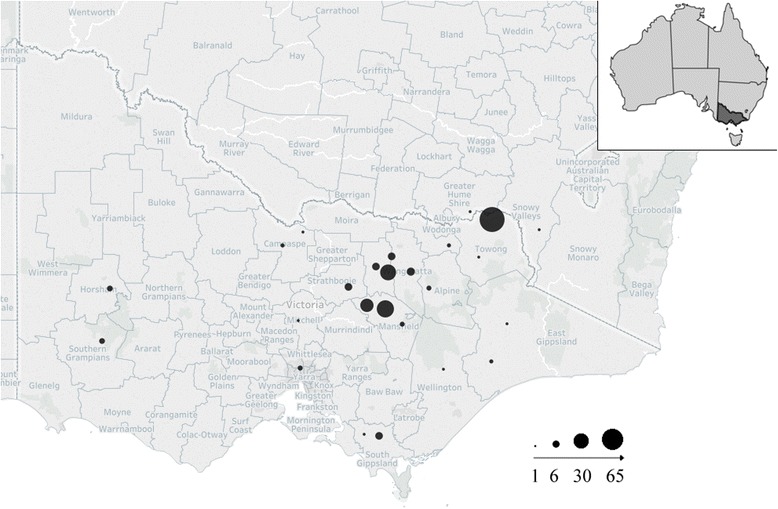
Origin of faecal samples of wild dogs, domestic dogs and foxes tested for the presence of *N. caninum* oocysts. Mark size is indicative of the number of samples collected per location

A single faecal sample per animal from wild dogs (*n* = 11) and red foxes (*n* = 188) was collected across Victoria, Australia (Fig. 1), by registered trappers and hunters during their normal hunting and trapping activities under Victorian legislation. A single faecal sample per animal from domestic dogs (*n* = 35) was collected by their respective owners or district veterinary officers, chilled (4 °C), and sent in via direct post for faecal flotation analysis within 7 days from collection, where possible. The geographical location of sample collection and the age, sex and breed of each animal was recorded, where possible. In addition, dog serum samples (*n* = 488) were collected following the conclusion of laboratory testing from two veterinary diagnostic laboratories (Australian Specialised Animal Pathology; the Veterinary Teaching Hospital, The University of Melbourne) located in Victoria, including five samples from dogs located outside of Victoria: three from New South Wales, one from South Australia and one from Tasmania. The home location, age, breed and sex of each animal was recorded for all samples. Of the non-Victorian samples, those from the mainland were all collected from sites adjacent to or straddling the state border.

### Faecal flotation

A flotation solution was made using 375 g/l of NaCl in water to create a solution with a specific gravity of 1.18–1.20 [15]. Faecal samples were examined microscopically for the presence of *N. caninum*-like oocysts (10–15 μm in diameter); oocysts of *N. caninum/Hammondia* spp. are shed unsporulated, unlike those of *Sarcocystis* spp., but, due to the time between excretion and examination, during which sporulation could have occurred, we included samples with unsporulated oocysts or with oocysts containing two sporocysts each with four sporozoites. The top layer of the flotation solution supernatant was transferred to a 15 ml tube, topped up with water and centrifuged at 1670× *g* for 5 min to create a pellet. The tube was stored at -20 °C until required for DNA extraction.

### DNA extraction and PCR

Stored faecal flotation samples from above (*n* = 179) were defrosted at room temperature (23 ± 2 °C) and centrifuged at 3220× *g* for 15 min and the pellet (< 200 mg) used for DNA isolation. All samples that were positive on flotation and a selection of negative samples were tested. DNA was extracted using a PowerSoil DNA Isolation Kit (MO BIO Laboratories, Carlsbad, CA, USA) following the manufacturer’s instructions with some modifications: the PowerBead Tubes were secured horizontally in a TissueLyser II (Qiagen, Hilden, Germany) and vortexed for 30 s at 30 Hz, and tubes were centrifuged at 10,000× *g* for 1 min for all steps requiring centrifugation. The DNA samples were eluted to 100 μl and stored at -20 °C until required for analysis by quantitative PCR.

Quantitative PCR (qPCR) was used to determine whether the *N. caninum* spp.-like oocysts observed in the faecal flotations were effectively *N. caninum* and not species in closely related coccidian genera like *Sarcocystis*, *Hammondia* and *Toxoplasma*. The primers and probe, targeting the Nc5 locus, a repetitive sequence present in the *N. caninum* genome, were based on those developed by Ghalmi et al. [16]. These primers and probes were checked for suitability using MEGA7 Alignment Explorer software (www.megasoftware.net). An error in the published reverse primer sequence was identified; a G at position 7. The updated primers and probe used in this study were NC5-550-F: 5′-GGG TGA ACC GAG GGA GTT G-3′; NC5-596-R: 5′-ACG TGA CGA ATG ACT AAC CAC AA-3′; NC5 probe: 5′-6FAM-AGC GGT GAG AGG TGG GAT ACG TGG-TAMRA-3′. A primer/probe mix was made with 10 μl of forward primer (10 μM), 10 μl of reverse primer (10 μM), 3 μl of probe (3 μM), and 77 μl of nuclease-free water. An 18S rRNA extraction control (TaqMan® Ribosomal RNA Control Reagents; Applied Biosystems, Foster City, CA, USA) was used to ensure correct extraction of sample DNA and RNA. Using AgPath-ID One-Step RT-PCR Reagents (Applied Biosystems), 5 μl of sample-extracted DNA was mixed with 12.5 μl of 2X RT-PCR buffer, 1 μl of primer/probe mix, 1 μl of enzyme, 0.375 μl of 18S rRNA primer/probe mix (1:1:1) and 5.125 μl of nuclease-free water, as per the manufacturer’s instructions. Samples then underwent the following thermocycling programme in a 7500 Fast Real-Time PCR System (Applied Biosystems): 1× (50 °C for 2 min), 1× (95 °C for 10 min), and 50× (95 °C for 15 s, 58 °C for 1 min) as per Ghalmi et al. [16]. All samples were run in duplicate. A no-template sample served as negative control, and *N. caninum* WA-K9 DNA in 1:10,000 and 1:100,000 dilutions was used as positive control (original concentration 100 ng/μl).

In addition, for flotation-positive samples, the apicomplexan D2 domain of the large subunit (LSU) rRNA gene was amplified using the CR1-CR2 primer pairs as published previously [17–19], MyTaq™Red Mix (Bioline Australia, Alexandria, NSW, Australia) and 2 μl of sample DNA in 25-μl reactions, with included controls, as described previously [20]. PCR products (~600 bp) were sequenced (Macrogen, Seoul, South Korea) and the resulting DNA sequences visually inspected for ambiguity and aligned with the homologous sequences from related cyst-forming coccidia (genera *Sarcocystis*,* Neospora*, *Toxoplasma*, *Cystoisospora*, *Hammondia*, *Besnoitia*) using CLC Main Workbench v6.9.1 (CLC bio, Aarhus, Denmark).

### Statistical analysis of faecal data

A backwards stepwise model-building process was used to develop a logistic regression model at the animal level, with variables retained if they were associated with *P* ≤ 0.05, derived from a likelihood-ratio test (RStudio packages nlme and lme4; RStudio, Boston, MA, USA). Potential risk factors evaluated were animal ‘type’ (fox, domestic dog, wild dog), sex (male, female), age (adolescent, adult, aged) and geographical location. Location was split into seven Victorian local government areas (LGAs): Benalla, Gippsland, Horsham, Hume, Mansfield, Towong and Wellington. Any locations situated outside of the boundaries of these LGAs were assigned to the geographically closest LGA. Results are presented as odds ratios and significance indicated using *P*-values.

### Competitive ELISA

Using the methodology previously described [21, 22], dog serum samples (*n* = 483) were screened for anti-*N. caninum* antibodies using a *N. caninum* Antibody Test Kit - cELISA (VMRD, Pullman, WA, USA) that was previously validated for use in cattle and partially validated for use in dogs [22–24]. Absorbance spectrophotometry was used to measure the percentage inhibition (%I) of colour change. The %I is the rate at which colour development is prevented, indicating a positive result. Sera were considered positive for anti-*N. caninum* antibodies when samples presented the manufacturer’s recommended %I cut-off of > 30%, a threshold validated for cattle.

### Indirect fluorescence antibody test

Competitive ELISA *N. caninum*positive (*n* = 39) and 40 negative (*n* = 41) dog serum samples were re-tested for the presence of anti-*N. caninum* antibodies using an indirect fluorescence test (IFAT). As in previous studies [11, 25, 26], IFAT tachyzoite *N. caninum* 12-well slides, FITC anti-canine serum, *N. caninum* canine positive serum and *N. caninum* canine negative serum (all from VMRD) were used according to the manufacturer’s instructions. Slides were examined with an Olympus BX60 microscope equipped to detect fluorescein isothiocyanate fluorescence (FITC, maximum excitation wavelength 490 nm; mean emission wavelength 530 nm). Under fluorescent light, the positive controls showed bright green banana-shaped tachyzoites of *N. caninum*, 4 to 5 μm in length. A sample was considered positive if complete peripheral tachyzoite labelling for anti-*N. caninum* antibodies was observed following a serum dilution of 1:50 (VMRD, manufacturer’s recommendation).

### Statistical analysis of serum data

Potential risk factors were assessed using univariable analyses and then, if significant (*P* ≤ 0.20), in separate multivariable models. A backwards stepwise model-building process was used to develop a logistic regression model at the animal level, with variables retained if *P* ≤ 0.05, derived from a likelihood-ratio test (RStudio). Biologically plausible interaction terms between main-effects variables were then considered for inclusion in the multivariable model. Summary measures of model goodness-of-fit included comparison of deviance to the degrees of freedom and Pearson *χ* ^2^ statistics. Results are presented as odds ratios and significance indicated using *P*-values, with statistical significance confirmed at *P* < 0.05. *P*-values of < 0.10 were indicated, where appropriate, to demonstrate where the result approached statistical significance.

Risk factors evaluated were sex (male, female), breed (categorised into small, medium, large; and categorised into the breed groupings of gundog, hound, utility, working, terrier, toy, non-sporting) and age (‘puppy’, ≤ 2 years old; ‘adolescent’, 2–5 years old; ‘adult’, 6–12 years old; ‘aged’, > 12 years old). In addition, the geographical location of each animal was categorised into either urban (metropolitan Melbourne and Geelong) or rural (all other locations). The urban category was further divided into 17 LGAs to evaluate this variable in more detail. The significance of selected individual breeds and their crosses (i.e. those with ≥ 10 animals in the study population: Jack Russell Terrier, Labrador Retriever, Border Collie, German Shepherd, Chihuahua, Maltese Terrier, Cavalier King Charles Spaniel, Staffordshire Bull Terrier and Kelpie), relative to all other breeds in the study population.

A Two-Graph Receiver Operating Characteristic (TG-ROC) was developed using the IFAT results as a reference to analyse the sensitivity and specificity of the ELISA at %I cut-off points increasing at 5% intervals from 0 to 100%.

## Results

### Presence of *N. caninum*-like oocysts in faecal samples

Of the 234 canid faecal samples, 18 samples (two of domestic dogs, one of a wild dog and 15 of foxes) contained oocysts matching the morphological characteristics of *N. caninum* spp.-like oocysts (Table 1). These oocysts measured approximately 11 (± 3) μm in diameter. Quantitative PCR was performed on 179 samples that either contained suspected oocysts (*n* = 18) or not (*n* = 161). While the 18S RNA extraction control could be amplified in all samples, none of the samples showed amplification of *N. caninum* genomic DNA. There were no significant associations between a faecal flotation-positive result for *N. caninum* and assessed potential risk factors, including animal type, age, sex or geographical location (*P* > 0.05).

**Table 1:** Presence of *Neospora caninum* oocysts in faecal samples of canids in Victoria, Australia

Origin of samples	*Neospora*-like oocysts observed in faeces				Positive Nc5 qPCR on oocysts
	Foxes	Domestic dogs	Wild dogs	Total (%)	
Benalla	2/51 (4%)	0/2 (0%)	1/5 (20%)	5	0/54
Gippsland	0/1 (0%)	0/8 (0%)	0/1 (0%)	0	0/3
Horsham	–	1/9 (11%)	–	11	0/3
Hume	–	1/6 (17%)	0/1 (0%)	14	0/6
Mansfield	4/55 (7%)	0/1 (0%)	–	7	0/45
Towong	7/73 (10%)	0/7 (0%)	–	9	0/53
Wellington	0/1 (0%)	0/2 (0%)	0/4 (0%)	0	0/6
Unknown location	2/7 (29%)	–	–	29	0/9
Total	15/188 (8%)	2/35 (6%)	1/11 (9%)	8	0/179

To determine the identity of the oocyst-like structures found in fox and dog faeces, we used a PCR reaction with primers that preferentially amplified DNA of cyst-forming coccidia (genera *Sarcocystis*, *Neospora*, *Toxoplasma*, *Cystoisospora*, *Hammondia* and *Besnoitia*) [17, 18, 27]. Twenty-four samples (of which five were from outside Victoria) containing oocyst-like structures were selected and 83% (20/24) produced a PCR amplicon of a size recognised as coccidian. Ten amplicons were of sufficient quantity for direct DNA sequencing. Seven PCR products produced a DNA sequence of satisfactory quality that, in each case, was near identical (> 99%) to *Sarcocystis tenella* D2 LSU rDNA (AF076899).

### Presence of anti-*N. caninum* antibodies in serum samples

Sera from domestic dogs were first assayed using cELISA for the detection of anti-*N. caninum* antibodies. Of the samples from Victorian dogs, 29.8% (144/483; 95% CI: 11.7–47.8%) tested positive for *N. caninum* antibodies using the manufacturer’s suggested %I cut-off of > 30%. Table 2 shows associations between animal level risk factors and a positive cELISA result for *N. caninum* in Victorian domestic dogs. Although breed, when divided into groups (Additional file 1: Table S1), was not significantly associated with *N. caninum* infection (*P* > 0.05), Jack Russell Terriers had a 22% higher risk of *N. caninum* infection (*P* = 0.05), regardless of geographical location, age or sex, when compared to the other breeds in the study population. Although the Border Collie breed was significantly associated with an cELISA-positive result in the univariable analysis, this could not be replicated in the multivariable analysis. The likelihood of an adolescent or adult dog being infected with *N. caninum* was 88% (*P* = 0.05) and 91% (*P* = 0.08), respectively, of the likelihood of an aged dog. However, puppy infection rates closely resembled (97%) those of aged dogs, the reference group. Furthermore, there was a 19% higher likelihood of *N. caninum* infection in rural versus urban domestic dogs (*P* = 0.10). When assessed at the LGA level, dogs from two LGAs, Brimbank (*P* = 0.01) and Boroondara (*P* = 0.04) (Additional file 2: Figure S1), had a lower likelihood of *N. caninum* infection relative to those from rural locations. The remaining risk factors, breed (small, medium and large) and sex, were not significantly associated with an ELISA-positive result for *N. caninum* (*P* > 0.05).

**Table 2:** Potential risk factors for Victorian domestic dogs tested positive for *Neospora caninum* antibodies by cELISA

Risk factor		β	SE (β)	*P*	OR	LCI	UCI
Breed	Jack Russell Terrier	0.20	0.10	0.05*	1.22	1.02	1.43
Age	Aged (12+ years)	Ref	–	–	Ref	–	–
	Adult (6–12 years)	-0.10	0.06	0.08	0.91	0.80	1.02
	Adolescent (2–5 years)	-0.13	0.07	0.05*	0.88	0.75	1.01
	Puppy (1–2 years)	-0.03	0.09	0.71	0.97	0.80	1.14
Geographical location	Rural	Ref	–	–	Ref	–	–
	Bayside	-0.01	0.18	0.97	0.99	0.64	1.35
	Boroondara	-0.27	0.13	0.04*	0.76	0.50	1.02
	Brimbank	-0.28	0.11	0.01*	0.75	0.54	0.97
	Cardinia	0.11	0.15	0.44	1.12	0.83	1.41
	Frankston	-0.17	0.17	0.31	0.84	0.51	1.17
	Glen Eira	-0.02	0.13	0.91	0.99	0.73	1.25
	Greater Dandenong	0.08	0.13	0.56	1.08	0.82	1.34
	Greater Geelong	-0.23	0.13	0.08	0.80	0.54	1.05
	Hobsons Bay	-0.13	0.13	0.31	0.87	0.62	1.13
	Macedon Ranges	-0.05	0.16	0.77	0.96	0.65	1.26
	Moreland	-0.13	0.19	0.50	0.88	0.51	1.25
	Melbourne City	-0.12	0.13	0.34	0.89	0.64	1.13
	Whitehorse	-0.22	0.15	0.14	0.80	0.51	1.09
	Whittlesea	-0.13	0.16	0.44	0.88	0.57	1.20
	Yarra	-0.20	0.15	0.19	0.82	0.51	1.12
	Yarra Ranges	-0.22	0.15	0.15	0.81	0.51	1.10

*Abbreviations:* *β* beta coefficient, *LCI* lower confidence interval, *OR* odds ratio, *Ref* reference point, *SE(β)* standard error of the beta co-efficient, *UCI* upper confidence interval

*P ≤ 0.05

**Fig. 2:**
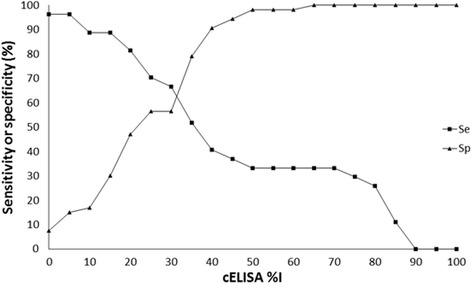
Two-graph receiver operating characteristic analysis of ELISA results against IFAT results. *Key*: squares: sensitivity (Se); triangles: specificity (Sp)

A selection of 39 cELISA-positive and 41 cELISA-negative samples was re-tested using IFAT to compare the *N. caninum* infection status of the dogs between these two tests. Among the (in total) 27 IFAT-positive dogs, only 18 were also found positive by cELISA, with 9 dogs presenting as cELISA false negatives. Among the 53 IFAT-negative dog sera, 23 tested positive using cELISA (Table 3). The sensitivity (Se) and specificity (Sp) of the cELISA at the 30%I cut-off were calculated to be 66.7 and 57.0%, respectively. The results of the cELISA and IFAT were analysed using TG-ROC to compare the Se and Sp of the cELISA at different %I cut-off points, increasing in intervals of 5% (Fig. 2). Se and Sp were equal (62%) at a %I cut-off of 31.5%.

**Table 3:** Overall comparison of IFAT and cELISA results for the detection of anti-*Neospora caninum* antibodies in serum of Victorian domestic dogs

		IFAT		
		+	–	Total
cELISA 30%I	+	18	23	41
	–	9	30	39
	Total	27	53	80

*Abbreviations: cELISA 30%I* competitive enzyme-linked immunosorbent assay 30% inhibition cut-off, *IFAT* indirect fluorescence antibody test

## Discussion

The calculated seroprevalence of *N. caninum* in Victoria determined by competitive enzyme-linked immunosorbent assay (cELISA) and indirect fluorescence antibody test (IFAT) was 29.8% (144/483; 95% CI: 11.7–47.8%) and 32.9% (27/80; 95% CI: 15.8–51.8%), respectively. The high IgG seroprevalence > 25% was unexpected, because a previous seroprevalence study on domestic dogs; urban, semiurban, rural, and euthanased at animal shelters, all domiciled in Melbourne, Victoria (*n* = 207) were tested using IFAT and the overall seroprevalence demonstrated only 5% (95% CI: 2–9%) of dogs positive for anti-*N. caninum* IgG antibodies in 1997 [14] and 9% (42/451; 95% CI: 6–12%) in Australia overall. A more recent study using sera from Aboriginal community dogs demonstrated *N. caninum* seroprevalence of 23.2% (45/194; 95% CI: 17.8–29.7%) and 20.6% (40/194; 95% CI: 15.5–26.9%) using cELISA and IFAT, respectively [22]. It was speculated, that the high *N. caninum* seroprevalence compared to urban dogs is caused by the scavenging behaviour of the examined dogs and consumption of wild animals suggestive of a sylvatic *N. caninum* cycle [5, 6, 22]. The even higher seroprevalence than Aboriginal community dogs detected in our study, suggests that opportunity for *N. caninum* infection is high in urban areas such as Melbourne, Victoria. The rather dramatic shift in seroprevalence from 5% to approximately 30% is potentially alarming, because of the consequences of clinical neosporosis in dogs [28, 29].

High seroprevalence in dog populations ultimately leads to increased vertical transmission from mother to foetus maintaining the parasite within the dog population [30]. In our study, puppies had a significantly greater seroprevalence than both adolescent and adult dogs; we speculate that thismay indicate a recent increase of seropositivity of pregnant bitches in the area, hence vertical transmission as well as post-weaning horizontal transmission and thatthe reason for the increased *N. caninum* seroprevalence could mean that, compared to the 1990s, the attitudes of dog owners to dogs’ diets have changed. In the past, dry processed food may have been more acceptable to pet owners, unlike now, where supplementing raw meat and bones into dogs’ diets is increasing. However, further studies are required to understand the reasons behind the 30% *N. caninum* seroprevalence. Horizontal (feeding infected meat) transmission of *N. caninum* is supported by increase in seroprevalance with age; however, there was greater seropositivity in rural than urban locations. There were clear differences within the metropolitan area of Melbourne that may reveal different attitudes and practices of dog owners. The finding that Jack Russell Terriers were 22% more likely to have *N. caninum* infection regardless of location, sex or age is valuable, but further studies are required to determine and confirm breed susceptibility to *N. caninum*.

In the current study, there were no confirmed *N. caninum* oocysts observed in the faeces of 179 Victorian canids. Faecal flotation can be used to isolate *N. caninum* oocysts shed in faeces and to determine the prevalence of neosporosis in a population [4, 22, 31]. However, *N. caninum* oocysts are morphologically indistinguishable from closely related genera, such as *Toxoplasma* and *Hammondia*. Therefore, genetic analysis of oocysts by PCR is required for confirmation [5, 25]. Exposure of both intermediate and definitive hosts to *N. caninum* is commonly evaluated using IFAT [16, 26] or ELISA [5, 32] to detect anti-*N. caninum* antibodies in serum. Despite its subjectivity, which is dependent on user observation of fluorescence, IFAT is currently considered the ‘gold standard’ for canine *N. caninum* serology, because it shows virtually no cross-reactivity with related protozoan parasites [2, 33, 34].

The *N. caninum*-like oocysts observed in faecal samples of several foxes, two domestic dogs and one wild dog were shown to be *Sarcocystis tenella*, a coccidian with a sheep-canid life-cycle, and known to be carried by foxes [25, 35], following further investigation where sufficient material was available. Identification of *N. caninum* oocysts is known to be difficult because of their morphological similarities to other coccidian genera. In addition, the shedding time-frame ranges from 1 day to several weeks [7, 36, 37], and the quantity of oocysts varies per sample [38, 39]. Despite the lack of *N. caninum* oocyst-shedding observed in this study, *N. caninum*-associated abortion in cattle is still occurring in Victoria with three confirmed abortion outbreaks this year and several more in the preceding years until 2012 (K. Moore, DEDJTR, pers. comm*.*). One sample from a domestic dog residing on a farm that was experiencing a *N. caninum*-associated outbreak of abortion at the time of sampling also tested negative. Epidemic abortion outbreaks are associated with point-source horizontal transmission to cattle [5, 40], which occur naturally via ingestion of canid-shed oocysts. The number of abortion outbreaks on Victorian farms from 2012 to 2016 indicate that, while shedding was not detected in this study, it is consistently occurring in the environment. The lack of obvious *N. caninum* shedding does not necessarily indicate its absence. Infected dogs may shed oocysts for short periods of time only, and factors influencing recurrence of shedding are poorly understood. Survival of oocysts in the environment is enhanced by a number of factors including immunosuppression [30]. Very little is known about the survival of *N. caninum* oocysts in the environment, however, it is assumed their environmental resistance is similar to that of *T. gondii* oocysts [30]. The year of this study may have had conditions that do not favour *N. caninum* oocyst-shedding or spread. There were indeed fewer confirmed *N. caninum*-associated abortions this year than in the previous few years (K. Moore, DEDJTR, pers. comm*.*).

The role of foxes in the transmission of *N. caninum* remains controversial, with one study identifying oocysts in faeces [31], but experimental transmission being unsuccessful [41]. The identification of *N. caninum* oocysts in fox faeces in a previous study may have been due to the ingestion of infective material containing tissue cysts and this may be the cause for positive amplification in some faecal samples [31]. Foxes are known definitive hosts of *S. tenella* as well as *Hammondia triffitae*, the oocysts of which resemble those of *N. caninum* [42].

IFAT is currently considered the gold standard for canine *N. caninum* serology [2, 34], and has been used since the early days of serological detection of this parasite [33]. IFAT is a subjective technique that relies on the experience of the individual analysing the samples, and is not quantifiable. The cELISA is a more recently developed method, and is objective and quantifiable. However, the cELISA kit used in this study was developed for use in cattle and may not use optimal reagents for detection of canine immunoglobulins. Partial validation of the cELISA kit found a naïve Se and Sp of 72 and 89%, respectively, and a corrected Se and Sp of 47 and 72%, respectively, at the 30%I cELISA cut-off for canine serum [24]. Similarly, King et al. [22] calculated a Se and Sp of 45 and 82%, respectively, at the 30%I cELISA cut-off. These numbers are slightly better than those calculated in the current study, but show that the low Se and Sp was expected.

It is time to reconsider the gold standard for *N. caninum* serology in canines because of the subjectivity of IFAT and the difficulty in using this technique for large numbers of samples. If ELISA is chosen as the new gold standard, it would need to be validated with a canine-specific antibody [43–45]. The above assessment of Se and Sp of the cELISA assumes that the IFAT had 100% Se and Sp. As indicated above, due to the subjective nature of this test, this assumption is likely false and statistical analyses that determine test Se and Sp in the absence of a gold standard (e.g. latent class analysis, Bayesian analysis) are required to determine the true Se and Sp of both the IFAT and cELISA in the diagnosis of *N. caninum* in this population.

## Conclusion

Effective prevention of spread of neosporosis in both bovine and canine populations can only be developed and implemented if transmission of *N. caninum* is fully understood. This study indicates that there is widespread infection among domestic canids despite a lack of shedding observed, and even farms with known *N. caninum* outbreaks have undetectable oocyst-shedding from the resident domestic dogs. The six-fold increase of seroprevalence in the canine population over 20 years indicates new transmission routes and behaviours of dog owners that require further elucidation. Our study demonstrates that data obtained 20 years ago can be misleading and current studies remain vital in understanding disease that cause significant burden to farm animals.

## Additional files

Additional file 1: Table S1. Evaluation of the association of potential risk factors for a domestic dog tested positive for *Neospora caninum* antibodies by univariable analysis. (DOCX 18 kb)

Additional file 2: Figure S1. Location map of Metropolitan Melbourne, Victoria, Australia showing local government area names. C, City Council; S, Shire Council. (PNG 130 kb)

## Abbreviations

%I: Percentage of inhibition
cELISA: Competitive enzyme-linked immunosorbent assay
CI: Confidence interval
IFAT: Indirect fluorescence antibody test
LGA: Local government area
qPCR: Quantitative polymerase chain reaction
