# Supplementary material for: High seroprevalance of Neospora caninum in dogs in Victoria, Australia, compared to 20 years ago
Source: Parasit Vectors. 2017 Oct 19;10:503. doi: 10.1186/s13071-017-2464-2 (PMC5649066; doi:10.1186/s13071-017-2464-2)
Supplement: Supplementary file 1 — Evaluation of the association of potential risk factors for a domestic dog tested positive for Neospora caninum antibodies by univariable analysis. (DOCX 18 kb) [file 13071_2017_2464_MOESM1_ESM.docx]

**Additional file 1: Table S1**. Association of potential risk factors for a domestic dog tested positive for *Neospora caninum* antibodies^1^.

| Risk factor | Subset | cELISA result | | *p* |
| --- | --- | --- | --- | --- |
|  |  | Negative | Positive |  |
| Breed Group | Unknown | 8 | 2 |  |
|  | Gundog | 66 | 27 |  |
|  | Hound | 20 | 7 |  |
|  | Non-sporting | 30 | 13 |  |
|  | Terrier | 48 | 29 |  |
|  | Toy | 92 | 32 |  |
|  | Utility | 29 | 14 |  |
|  | Working | 46 | 20 | 0.77 |
| Breed |  |  |  |  |
| Beagle | No | 329 | 141 |  |
|  | Yes | 8 | 3 | 0.85 |
|  |  |  |  |  |
| Border Collie | No | 324 | 142 |  |
|  | Yes | 13 | 2 | 0.15* |
|  |  |  |  |  |
| Cavalier King Charles Spaniel | No | 329 | 141 |  |
|  | Yes | 8 | 3 | 0.85 |
|  |  |  |  |  |
| Chihuahua | No | 329 | 138 |  |
|  | Yes | 8 | 6 | 0.28 |
|  |  |  |  |  |
| German Shepherd | No | 329 | 138 |  |
|  | Yes | 8 | 6 | 0.28 |
|  |  |  |  |  |
| Jack Russell Terrier | No | 327 | 134 |  |
|  | Yes | 10 | 10 | 0.05* |
|  |  |  |  |  |
| Kelpie | No | 327 | 139 |  |
|  | Yes | 10 | 5 | 0.77 |
|  |  |  |  |  |
| Labrador/Golden Retriever | No | 299 | 127 |  |
|  | Yes | 38 | 17 | 0.86 |
|  |  |  |  |  |
| Maltese Terrier | No | 294 | 130 |  |
|  | Yes | 43 | 14 | 0.35 |
|  |  |  |  |  |
| Poodle | No | 327 | 138 |  |
|  | Yes | 10 | 6 | 0.50 |
|  |  |  |  |  |
| Shih Tzu | No | 326 | 142 |  |
|  | Yes | 11 | 2 | 0.25 |
|  |  |  |  |  |
| Staffordshire Bull Terrier | No | 323 | 134 |  |
|  | Yes | 14 | 10 | 0.20 |
|  |  |  |  |  |
| Location (local government area) | Bayside | 5 | 4 |  |
|  | Boroondara | 24 | 6 |  |
|  | Brimbank | 125 | 28 |  |
|  | Cardinia | 8 | 10 |  |
|  | Frankston | 8 | 3 |  |
|  | Glen Eira | 16 | 13 |  |
|  | Greater Dandenong | 14 | 16 |  |
|  | Greater Geelong | 25 | 7 |  |
|  | Hobsons Bay | 20 | 10 |  |
|  | Macedon Ranges | 8 | 6 |  |
|  | Melbourne | 24 | 13 |  |
|  | Moreland | 5 | 3 |  |
|  | Rural | 11 | 9 |  |
|  | Whittlesea | 13 | 4 |  |
|  | Yarra | 11 | 4 |  |
|  | Yarra Ranges | 13 | 4 | < 0.01* |
|  |  |  |  |  |
| Animal Age | Puppy | 26 | 14 |  |
|  | Adolescent | 78 | 27 |  |
|  | Adult | 173 | 66 |  |
|  | Aged | 59 | 37 | 0.15* |
|  |  |  |  |  |
| Animal Sex | Male | 167 | 75 |  |
|  | Female | 171 | 68 | 0.54 |

* *p* value < 0.20 (significant at univariable level)

^1^The association was evaluated using univariate analysis.
