# Supplementary figures and images for: High seroprevalance of Neospora caninum in dogs in Victoria, Australia, compared to 20 years ago
Source: Parasit Vectors. 2017 Oct 19;10:503. doi: 10.1186/s13071-017-2464-2 (PMC5649066; doi:10.1186/s13071-017-2464-2)

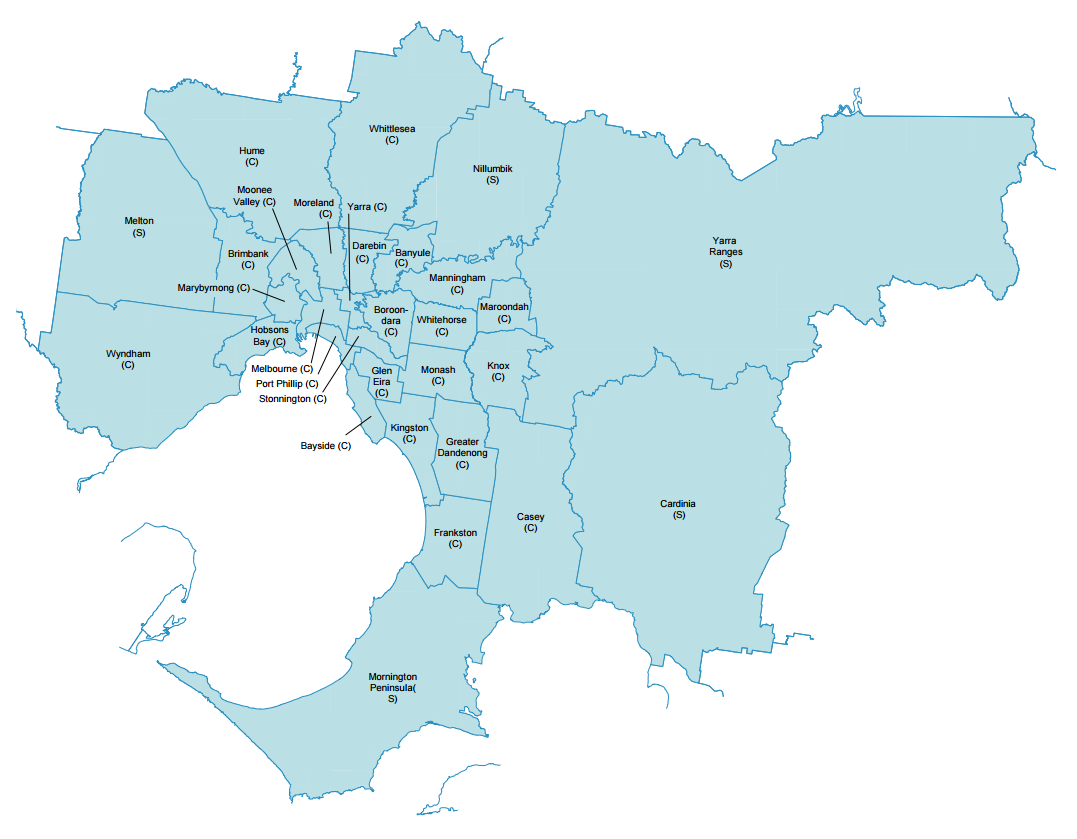

Supplement: Supplementary file 2 — Location map of Metropolitan Melbourne, Victoria, Australia showing local government area names. C, City Council; S, Shire Council. (PNG 130 kb) [file 13071_2017_2464_MOESM2_ESM.png]
